# Relevance of psychiatry in dermatology: Present concepts

**DOI:** 10.4103/0019-5545.70992

**Published:** 2010

**Authors:** K. H. Basavaraj, M. A Navya, R. Rashmi

**Affiliations:** Department of Dermatology, Venereology and Leprosy, JSS Medical College, JSS University, Mysore - 570 015, Karnataka, India

**Keywords:** Liaison therapy, mind, psychodermatology, quality of life, stress

## Abstract

Skin is an organ that has a primary function in tactile receptivity and reacts directly upon emotional stimuli. Dermatological practice involves a psychosomatic dimension. A relationship between psychological factors and skin diseases has long been hypothesized. Psychodermatology addresses the interaction between mind and skin. It is divided into three categories according to the relationship between skin diseases and mental disorders. This article reviews different dermatological conditions under each of the three categories namely psychosomatic disorders, dermatological conditions due to primary and secondary psychiatric disorders. Dermatological conditions resulting from psychiatric conditions like stress/depression and those caused by psychiatric disorders are discussed. This review intends to present the relationship between the ‘skin’ and the ‘mind’ specifically from the dermatology point of view. The effects on the quality of life as a result of psychodermatological conditions are highlighted. A multidisciplinary approach for treatment from both dermatologic and psychiatric viewpoints are suggested.

## INTRODUCTION

Skin has a special place in psychiatry with its responsiveness to emotional stimuli and ability to express emotions such as anger, fear, shame and frustration, and by providing self-esteem, the skin plays an important role in the socialization process, which continues from childhood to adulthood.[1] The relationship between skin and the brain exists due to more than a fact, that the brain, as the center of psychological functions, and the skin, have the same ectodermal origin and are affected by the same hormones and neurotransmitters.[2] Psychodermatology describes an interaction between dermatology and psychiatry and psychology. The incidence of psychiatric disorders among dermatological patients is estimated at about 30 to 60%.[3] Psychiatry is more focused on the ‘internal’ non-visible disease, and dermatology is focused on the ‘external’ visible disease. Connecting the two disciplines is a complex interplay between neuroendocrine and immune systems that has been described as the NICS, or the neuro-immuno-cutaneous system. The interaction between nervous system, skin and immunity has been explained by release of mediators from NICS.[4] It has been reported that psychologic stress perturbs epidermal permeability barrier homeostasis, and it may act as precipitant for some inflammatory disorders like atopic dermatitis and psoriasis.[5] Dermatologists have stressed the need for psychiatric consultation in general, and psychological factors may be of particular concern in chronic intractable dermatologic conditions, such as eczema, prurigo and psoriasis.[6,7] Patients with psychocutaneous disorders frequently resist psychiatric referral, and the liaison among primary care physicians, psychiatrists and dermatologists can prove very useful in the management of these conditions. Thus consideration of psychiatric and psychosocial factors is important both for the management of psychodermatologic disorders and for some aspects of secondary and tertiary prevention of a wide range of dermatologic disorders.[8] Regardless of psychiatric morbidity, skin diseases can greatly affect patients’ quality of life.[9] The drugs used in the treatment of dermatological diseases such as steroid and retinoid may lead to psychiatric symptoms.[10] Not surprisingly, a relationship between psychological factors and skin diseases has long been hypothesized. There is a common opinion that many cases of skin disease are caused by psychological stress, or are related to certain personality traits, or represent a complication of a psychiatric disorder. Although the dermatologists awareness of the problem is increasing,[11] co-occurring mental disorders go often unrecognized and are believed to be less frequent than they actually are in many skin conditions. There is a need for a biopsychosocial approach to patients with skin disease.[12,13] Liaison therapy enables multidisciplinary approach with the cooperation of psychiatric and dermatologic terms and simultaneous diagnostic procedures and treatment of patients with psychodermatologic disorders.[14]

## CLASSIFICATION

Although there is no single universally accepted classification system of psychocutaneous disorders and many of the conditions are overlapped into different categories, the most widely accepted system is that devised by Koo and Lee.[15]

**Figure 1:**
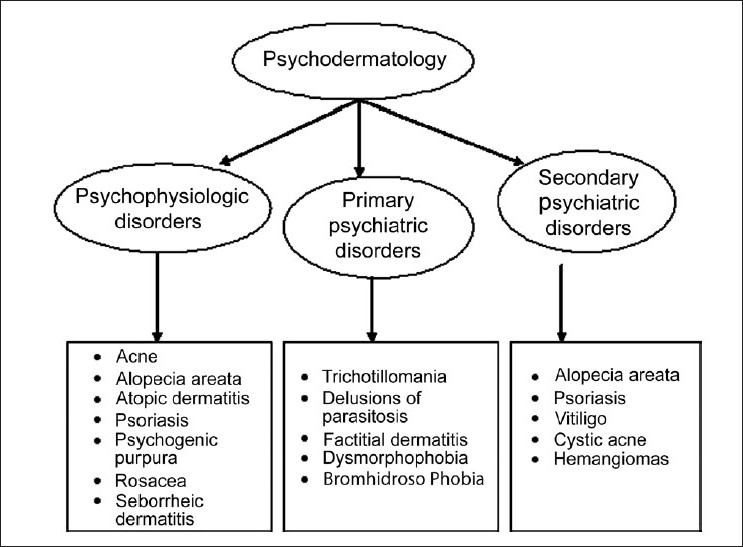
Common psychodermatology disorders

Psychodermatology is divided into three categories according to the relationship between skin diseases and mental disorders [Figure 1]: 1) Psychophysiologic (psychosomatic) disorders caused by skin diseases triggering different emotional states (stress), but not directly combined with mental disorders (psoriasis, eczema); 2) primary psychiatric disorders responsible for self-induced skin disorders (trichotillomania) and 3) secondary psychiatric disorders caused by disfiguring skin (of ichthyosis, acne conglobata, vitiligo), which can lead to states of fear, depression or suicidal thoughts.[8]

### Psychophysiologic (Psychosomatic) disorders

Here psychiatric factors are instrumental in the etiology and course of skin conditions. The skin disease is not caused by stress but appears to be precipitated or exacerbated by stress.[15]

### Psoriasis

Psoriasis is a relatively common, chronic and inflammatory and hyperproliferative skin disease that occasionally requires systemic therapy.[16] Stress has long been reported to trigger psoriasis.[17] Psoriasis is associated with a variety of psychological difficulties, including poor self-esteem, sexual dysfunction, anxiety, depression and suicidal ideation. Psoriasis is associated with substantial impairment of health-related quality of life (HRQOL), negatively impacting psychological, vocational, social and physical functioning.[18] The most common psychiatric symptoms attributed to psoriasis include disturbances in body image and impairment in social and occupational functioning.[19] Quality of life may be severely affected by the chronicity and visibility of psoriasis as well as by the need for lifelong treatment. Five dimensions of the stigma associated with psoriasis have been identified: (1) Anticipation of rejection, (2) feelings of being flawed, (3) sensitivity to the attitudes of society, (4) guilt and shame and (5) secretiveness.[20] Depressive symptoms and suicidal ideation was frequently associated in psoriasis.[21–26] In general, psychological factors, including perceived health, perceptions of stigmatization and depression are stronger determinants of disability in patients with psoriasis than are disease severity, location and duration.[27] In a recent prospective study of patients with psoriasis,[28] the frequency of psychiatric disturbance decreased with improvement in the clinical severity and symptoms of psoriasis. The emotional effects and functional impact of the disease are not necessarily proportionate to the clinical severity of psoriasis.[29]

### Atopic dermatitis

The onset or exacerbation of atopic dermatitis often follows stressful life events.[30] Symptom severity has been attributed to interpersonal and family stress, and problems in psychosocial adjustment and low self-esteem have been frequently noted.[31,32] Adults with atopic dermatitis are more anxious and depressed compared with clinical and healthy control groups.[33,34] Children with atopic dermatitis have higher levels of emotional distress and more behavioral problems than healthy children or children with minor skin problems.

### Psychosocial morbidity in atopic dermatitis

Psychological stress may be an acquired factor affecting the expression of atopic dermatitis.[35] Atopic individuals with emotional problems may develop a vicious cycle between anxiety/depression and dermatologic symptoms. In one direction, anxiety and depression are frequent consequences of the skin disorder. The misery of living with atopic dermatitis may have a profoundly negative effect on health-related quality of life (HRQOL) of children and their families. Teasing and bullying by children and embarrassment by adults and children can cause social isolation and school avoidance. The social stigma of a visible skin disease, frequent visits to doctors and the need to constantly apply messy topical remedies all add to the burden of disease. Lifestyle restrictions in more severe cases can be significant, including limitations on clothing, staying with friends, owning pets, swimming or playing sports. The impairment of quality of life caused by childhood atopic dermatitis has been shown to be greater than or equal to that of asthma or diabetes.[36]

## URTICARIA

Severe emotional stress may exacerbate preexisting urticaria.[37] Increased emotional tension, fatigue, and stressful life situations may be primary factors in more than 20% of cases and are contributory in 68% of patients. Difficulties with expression of anger and a need for approvals from others are also common.[38,39] Patients with this disorder may have symptoms of depression and anxiety, and the severity of pruritus appears to increase as the severity of depression increases.[40,41] Cold urticaria may be associated with hypomania during winter and recurrent idiopathic urticaria with panic disorder.[42]

### Acne excoriée

The psychosocial effect of acne was first recognized in 1948, when Sulzberger and Zaidens wrote, ’There is no single disease which causes more psychic trauma and more maladjustment between parents and children, more general insecurity and feelings of inferiority, and greater sums of psychic assessment than does acne vulgaris’.[43] Acne has a demonstrable association with depression and anxiety and psychiatric comorbidity of acne excoriée includes body image disorder, depression, anxiety, obsessive-compulsive disorder (OCD), delusional disorders, personality disorders and social phobias.[44–47] It has been reported that young men with severe scarring acne are at particular risk of depression and suicide.[48] Interesting gender differences have been observed in this disease. In men, self-excoriation is exacerbated by a coexisting depression or anxiety, while in women this behavior may be a manifestation of immature personality and serve as an appeal for help.[49]

### Primary psychiatric disorders

Primary psychiatric disorders are encountered less often than psychophysiologic disorders. These disorders have received little emphasis in the psychiatry or dermatology literature, even though they may be associated with suicide and unnecessary surgical procedures. Most of these disorders occur in the context of somatoform disorder, anxiety disorder, factitious disorder, impulse-control disorder or eating disorder.[50]

#### Psychogenic excoriation

Psychogenic excoriation occurs in 2% of dermatology patients mostly in women. It is an uncommon psychodermatological condition, which responds well to serotonin reuptake inhibitors and behavioral therapy. It is characterized by excessive scratching or picking of the skin. The lesions are usually found on face, upper limbs and upper back.[51] It is a chronic disorder with a high rate of psychiatric comorbidity. Major depressive syndrome was the most common psychiatric disorder found in the PE group.[52]

#### Delusions of parasitosis

The most common form of monosymptomatic hypochondriacal psychosis encountered among patients with skin problems is called delusions of parasitosis.[53] Delusional parasitosis is a syndrome in which the patient has the false belief that he is infested by parasites or organism; and they often elaborate on how these organisms reproduce, move and spread under their skin, or even exit the skin. It may occur as the sole psychologic disturbance, or it may be associated with an underlying psychiatric disorder or physical illness.[54] The psychiatric differential diagnosis include schizophrenia, psychotic depression, psychosis in patients with florid mania or drug-induced psychosis, and formication without delusion, in which the patient experiences crawling, biting and stinging sensations without believing that they are caused by organisms.[55] Patients with delusion of parasitosis often present with the matchbox sign, in which small bits of excoriated skin, debris or unrelated insects or insect parts are brought in matchboxes or other containers as a proof of infestation.[21]

#### Trichotillomania

Trichotillomania, according to the dermatologic use of the word, is a condition in which a person pulls out his or her own hair. The psychiatric definition of trichotillomania requires the presence of ‘impulsivity’.[56] The most common underlying psychopathology is obsessive-compulsive behavior.[57] Other possible underlying psychiatric disorders are: Reaction to stress, anxiety, depression, behavioral disorder, mental retardation and delusions in which the patient pulls out his or her hair based on a delusional belief that something in the roots needs to be ‘dug out’ so the hair can grow normally. This latter, rare condition is called ‘trichophobia’.[21] Childhood trauma and emotional neglect may play a role in the development of this disorder.[58] The patients experience an increasing sense of tension immediately before an episode of hair pulling and when attempting to resist the behavior, they feel relieved of tension and sometimes a feeling of gratification after hair pulling.[59]

#### Obsessive-compulsive disorder

Patients usually present to dermatologists because of skin lesions resulting from scratching, picking, and other self-injurious behaviors. They typically have an increased level of psychiatric symptomatology compared with age and sex matched controls taken from the general population of dermatology patients, and many patients experience negative stigmatization in their daily life.[60–62] Common behaviors include compulsive pulling of scalp, eyebrow, or eyelash hair; biting of the nails and lips, tongue and cheeks; and excessive hand washing. It has been found that OCD in child and adolescent dermatology patients most commonly presents as trichotillomania, onychotillomania and acne excoriée.[63]

#### Dysmorphophobia

This condition is also called body dysmorphic disorder or dermatological non-disease.[64] Patients with this condition are rich in symptoms but poor in signs of organic disease. Self-reported ‘complaints’ or ‘concerns’ usually occur in three main areas: Face, scalp and genitals. Facial symptoms include excessive redness, blushing, scarring, large pores, facial hair and protruding or sunken parts of face. Other symptoms are hair loss, red scrotum, urethral discharge and herpes and AIDS phobia. Strategies to relieve the anxiety due to the perceived defects may include camouflaging the lesions, mirror checking, comparison of ‘defects’ with the same body parts on others, questioning/reassurance seeking, mirror avoidance and grooming to cover up ‘defects’.[65] Women are more likely than men to be preoccupied with the appearance of their hips or their weight, to pick their skin, to camouflage defects with makeup and to have comorbid bulimia nervosa. Men are more likely than women to be preoccupied with body build, genitals and hair thinning and to be unmarried and to abuse alcohol.[66] Patients with body image disorders, especially those involving the face, may be suicidal.[67] Associated comorbidity in dysmorphophobia may include depression, impairment in social and occupational functioning, social phobias, OCD, skin picking, marital difficulties and substance abuse.[68,69]

#### Dermatitis artifacta (Factitial dermatitis)

This is an artifactual skin disease caused entirely by the actions of the fully aware patient on the skin, hair, nails or mucosa, with no rational motive for this behavior. The condition is more common in women than in men (3 : 1 to 20 : 1).[70] The lesions are usually bilaterally symmetrical, within easy reach of the dominant hand, and may have bizarre shapes with sharp geometrical or angular borders, or they may be in the form of burn scars, purpura, blisters and ulcers. Erythema and edema may be present. Patients may induce lesions by rubbing, scratching, picking, cutting, punching, sucking or biting or by applying dyes, heat or caustics. Some patients inject substances, including feces and blood. Reported associated conditions include OCD, borderline personality disorder, depression, psychosis and mental retardation,[71,72] and most of the patients with factitious dermatitis have some sort of personality disorder and they often use some means to damage his or her own skin, such as burning cigarettes, chemicals or sharp instruments.[73]

#### Psychogenic pruritus

In this disorder, there are cycles of stress leading to pruritus as well as of the pruritus contributing to stress. Psychologic stress and comorbid psychiatric conditions may lower the itch threshold or aggravate itch sensitivity.[74] Stress liberates histamine, vasoactive neuropeptides and mediators of inflammation, while stress-related hemodynamic changes (e.g., variation in skin temperature, blood flow and sweat response) may all contribute to the itch-scratch-itch cycle.[75] Psychogenic pruritus has been noted in patients with depression, anxiety, aggression, obsessional behavior and alcoholism. The degree of depression may correlate with pruritus severity.[76]

### Secondary psychiatric disorders

This category includes patients who have emotional problems as a result of having skin disease. The skin disease in these patients may be more severe than the psychiatric symptoms, and, even if not life-threatening, it may be considered ‘life-ruining’.[21] Symptoms of depression and anxiety, work-related problems and impaired social interactions are frequently observed.[77]

#### Alopecia areata

The role of psychological factors in the pathogenesis of alopecia areata (AA) has long been the subject of debate.[78] The influence of psychologic factors in the development, evolution and therapeutic management of alopecia areata is well established. Acute emotional stress may precipitate alopecia areata, perhaps by activation of overexpressed type 2b corticotropin-releasing hormone receptors around the hair follicles, and lead to intense local inflammation.[79] Release of substance P from peripheral nerves in response to stress has also been reported, and prominent substance P expression is observed in nerves surrounding hair follicles in alopecia areata patients.[80] Substance P degrading enzyme neutral endopeptidase has also been strongly expressed in affected hair follicles in the acute-progressive as well as the chronic-stable phase of the disorder. Comorbid psychiatric disorders are also common and include major depression, generalized anxiety disorder, phobic states and paranoid disorder.[81]

#### Vitiligo

Vitiligo is a specific type of leukoderma characterized by depigmentation of the epidermis. In some studies, patients with vitiligo have been found to have significantly more stressful life events compared with controls, suggesting that psychologic distress may contribute to onset.[82] Links between catecholamine-based stress, genetic susceptibility and a characteristic personality structure have been postulated.[83] Psychiatric morbidity is typically reported in approximately one-third of patients,[82] but, in one study, 56% of the sample had adjustment disorder and 29% had depressive disorders.[84] Patients with vitiligo are frightened and embarrassed about their appearance, and they experience discrimination and often believe that they do not receive adequate support from providers.[85] Younger patients and individuals in lower socioeconomic groups show poor adjustment, low self-esteem and problems with social adaptation.[86,87] Most patients with vitiligo report a negative impact on sexual relationships and cite embarrassment as the cause.[88]

## CONCLUSION

Psychodermatologic disorders are conditions involving interaction between the mind and the skin. They fall into three categories; psychosomatic, primary psychiatric disorders and secondary psychiatric disorders. Atopic dermatitis, eczema, urticaria, psoriasis, herpes simplex, alopecia areata, rosacea, etc are regarded among dermatological psychosomatic disorders with psychogenic manifestation/exacerbation. It is suggested to use a biopsychological model, which takes into account the psychological (e.g. psychiatry comorbidity such as major depression and the impact of skin disorder on the psychological aspects of quality of life) and social (e.g. impact upon social and occupational functioning) factors, in addition to the primary dermatologic factors, in the management of the disease. The treatment of psychodermatological disorders should be carried out through the liaison therapy, which enables multidisciplinary approach, including family physician, dermatologist, psychiatrist and psychologist. It is very important to educate dermatologists in the diagnostic procedures and therapy of psychiatric disorders, which sometimes coexist with the skin disease. Majority of psychodermatological disorders can be treated with cognitive-behavioral psychotherapy, psychotherapeutic stress-and-anxiety-management techniques and psychotropic drugs. Psychopharmacologic treatment includes anxiolytics, antidepressants, anti-psychotics and mood stabilizer. The cooperation of the dermatologist and a psychiatrist in order to increase the life quality of the patients is of utmost importance. A dermatologist’s lack of knowledge on the psychiatric morbidity rates in dermatological diseases may delay the diagnosis of psychiatric condition and hinder the treatment, and hence establishment of separate psychodermatology units and multicenter research about the relationship of skin and psyche is necessary in the form of prospective case-controlled studies, and multisite therapeutic trials can provide more insight into this interesting and exciting field of medicine. The management of psychodermatologic disorders requires evaluation of the skin manifestation and the social, familial and occupational issues underlying the problem. Once the disorder has been diagnosed, management requires a dual approach, addressing both dermatologic and psychologic aspects. A mutual, respectful collaboration between dermatologists and mental health professionals might be of help for many psychiatric patients. Therefore, understanding of biopsychosocial approaches and liaison approach involving general practice, psychiatrist, dermatologist and psychologist treatment in this field is essential.
